# Accelerated Identification and Preliminary Validation of a Pathogenic Missense Variant in the *L1CAM* Gene in a Pregnant Woman With Sonographic Anomalies Using AlphaMissense

**DOI:** 10.1002/mgg3.70169

**Published:** 2025-12-21

**Authors:** Zhihui Wang, Xuna Shen, Chenyang Xu, Rongyue Wang, Chendi Teng, Yanbin He, Weiyan Wu, Xutao Hong

**Affiliations:** ^1^ Department of Gynecology and Obstetrics Wenzhou Central Hospital Zhejiang China; ^2^ Department of Central Laboratory Wenzhou Central Hospital Zhejiang China; ^3^ Department of Obstetrics and Gynecology The Second Affiliated Hospital and Yuying Children's Hospital of Wenzhou Medical University Wenzhou China; ^4^ Department of Radiology Wenzhou Central Hospital Zhejiang China; ^5^ Zhejiang Key Laboratory of Digital Technology in Medical Diagnostics Hangzhou China; ^6^ Yikon Genomics Co., Ltd Shanghai China

**Keywords:** AlphaFold, AlphaMissense, exome sequencing, hydrocephalus, *L1CAM*, prenatal diagnosis

## Abstract

**Background:**

Prenatal diagnosis of X‐linked hydrocephalus caused by variants in the *L1CAM* gene is often complicated by the identification of Variants of Uncertain Significance (VUSs). This study showcases an accelerated diagnostic workflow using artificial intelligence (AI) to rapidly interpret a novel missense variant for a family with a history of the disorder.

**Methods:**

We performed exome sequencing (ES) on a male fetus with significant sonographic brain anomalies from a 29‐year‐old pregnant woman. To efficiently analyze the resulting VUSs, we used the AI tool AlphaMissense to predict their pathogenicity and prioritize them for validation. The top candidate variant was then assessed via Sanger sequencing for co‐segregation across eight maternal relatives. The structural impact of the mutation was visualized using the AlphaFold 3 model.

**Results:**

Exome sequencing identified four VUSs. AlphaMissense predicted only one, *L1CAM* c.1228C>G (p.His410Asp), as ‘likely pathogenic’. Subsequent Sanger sequencing confirmed that this variant co‐segregated perfectly with the disease phenotype in the family. Based on this strong genetic evidence, the variant was reclassified from a VUS to ‘Likely Pathogenic’. Structural modeling revealed that the p.His410Asp substitution disrupts a critical salt bridge, likely compromising protein stability.

**Conclusion:**

Our two‐step approach—using AI for rapid VUS prioritization followed by targeted Sanger validation—proved to be a highly efficient strategy. It provided a definitive and clinically actionable diagnosis that facilitated genetic counseling and enabled the family to pursue Preimplantation Genetic Testing (PGT). This workflow significantly enhances the power of genomic testing in the prenatal setting.

## Graphical abstract

This study demonstrates an efficient workflow for identifying pathogenic variants in prenatal diagnosis. We utilized exome sequencing coupled with the AI tool AlphaMissense to prioritize Variants of Uncertain Significance (VUSs) in a family with X‐linked hydrocephalus. The identified *L1CAM* variant (p.His410Asp) was validated via Sanger sequencing and structural modeling using AlphaFold 3, revealing a disrupted salt bridge. This approach facilitates rapid diagnosis and clinical management, such as Preimplantation Genetic Testing (PGT).

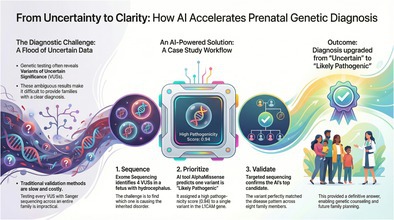

## 1 Introduction

Hydrocephalus is a significant birth defect, occurring in approximately 9.2 per 10,000 live births in China (Huang et al. 2018). Hereditary is often associated with pathogenic variants in the *L1CAM* gene (OMIM: 308840), which follows an X‐linked inheritance pattern. Detecting pathogenic variants is vital in prenatal diagnostics, informing clinical management and family planning. Early identification can facilitate specialized care, improving outcomes for both mother and child. However, testing may also reveal variants of uncertain significance (VUSs), often amino acid substitutions, which pose challenges in clinical genetics (McInnes et al. 2021). Despite guidelines from the American College of Medical Genetics and Genomics (ACMG) (Richards et al. 2015), novel missense variants often pose challenges, with approximately 75%–92% classified as VUS. This complicates clinical decision‐making and hinders effective treatment strategies.

Exome sequencing (ES) of all family members can lead to high costs and efficiency challenges. Furthermore, validating every VUS with Sanger sequencing in extended families is inefficient.

The application of artificial intelligence in healthcare is rapidly advancing. In 2021, DeepMind's AlphaFold was developed to accurately predict protein structures (Jumper et al. 2021), allowing researchers to move from the cumbersome process of protein structure analysis to more efficient structure validation. AlphaMissense is a computational tool predicting the pathogenicity of missense variants using deep learning and genomic databases. It provides insights into their clinical impact, with predictions aligning approximately 90% with ClinVar (Cheng et al. 2023), underscoring its reliability.

In this study, we first identified VUS variants through ES of the proband and used AlphaMissense to predict pathogenicity, refining our focus for validation. For likely pathogenic variants, we employed Sanger sequencing to confirm changes in family members, thus reducing costs and increasing efficiency. This study aims to explore the application of AlphaFold and AlphaMissense in identifying and validating pathogenic missense VUS variants, enhancing detection efficiency in clinical settings and improving prenatal diagnostics for at‐risk families.

## 2 Materials and Methods

### 2.1 Ethical Compliance

This study was approved by the Ethics Committee of our institution, and written informed consent was obtained from all participating family members.

### 2.2 Subjects

A 29‐year‐old pregnant woman, at 23 weeks of gestation, presented to our prenatal diagnosis center after ultrasound examinations at another facility indicated bilateral ventriculomegaly. The couple was non‐consanguineous and of Han Chinese ethnicity. Her medical history included a prior pregnancy where an ultrasound at 23 weeks revealed fetal hydrocephalus and agenesis of the corpus callosum, leading to induced labor without genetic testing. The patient reported a family history of intellectual disabilities, with two affected maternal uncles.

**FIGURE 1:**
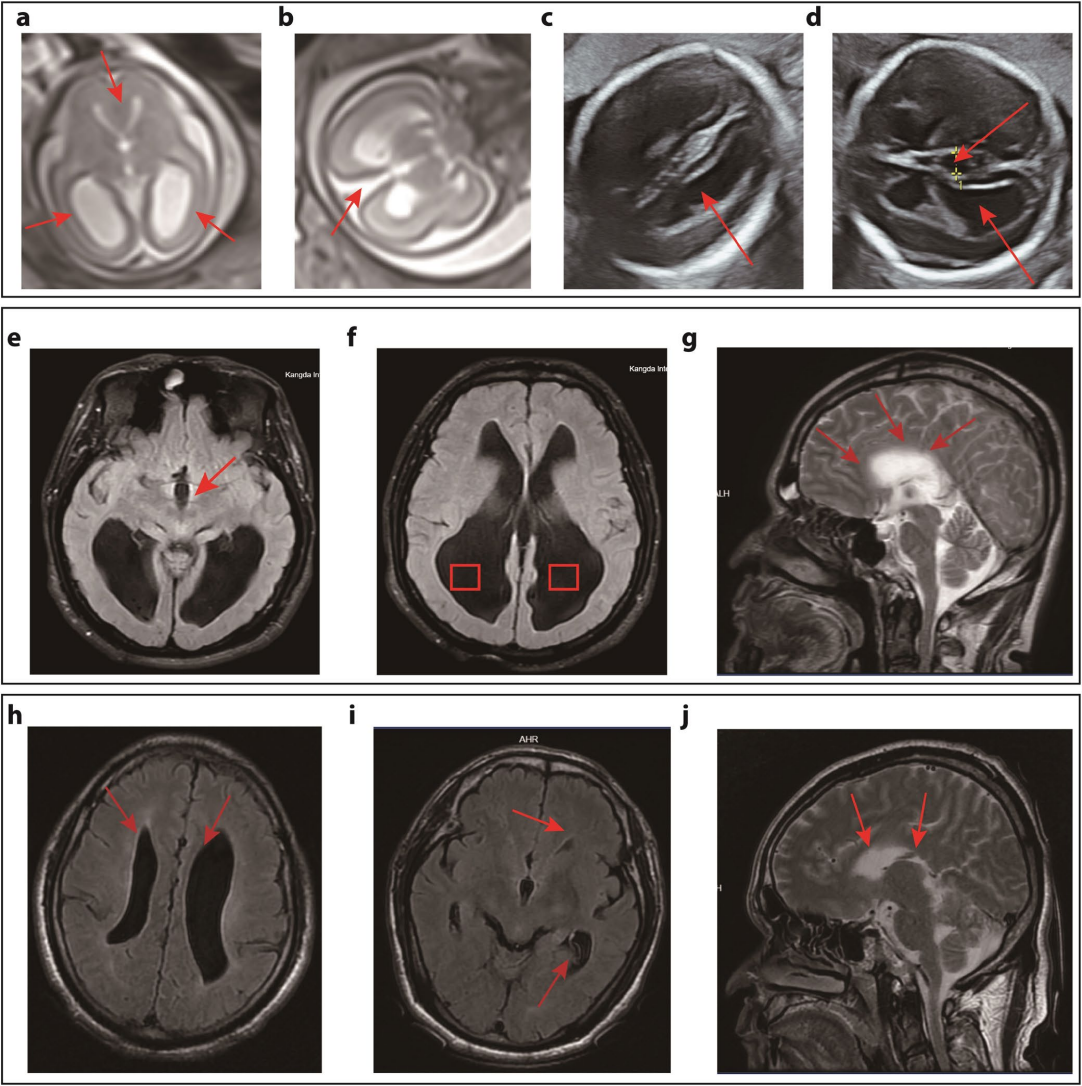
Neuroimaging of the proband and affected relatives. (a–d) Neuroimaging of the male proband at 23 weeks gestation. Fetal MRI (a, b) and ultrasound (c, d) show key features of *L1CAM*‐associated hydrocephalus, including agenesis of the corpus callosum and severe ventriculomegaly. (e–j) Brain MRIs of two affected maternal uncles (Uncle 1: e–g; Uncle 2: h–j) display characteristic findings, such as an absent corpus callosum and enlarged, malformed ventricles.

Our reassessment via prenatal ultrasound of the current fetus (the proband) revealed significant findings: Genetic testing from the amniotic fluid had confirmed the fetus was male. The anterior horns and body of the fetal lateral ventricles showed lateral expansion, with posterior horns dilated to 14 mm, resembling a “teardrop” shape. The third ventricle was elevated at 5 mm, suggesting possible agenesis of the corpus callosum. Fetal brain MRI confirmed fusion of the fetal forebrain, enlarged ventricles, and partial absence of the corpus callosum (Figure 1a–d).

During pre‐test genetic counseling, it was noted that the woman has two normal maternal aunts and two intellectually disabled maternal uncles, who were also referred for MRI evaluation. A strategy was devised to extract genomic DNA from the fetal amniotic fluid and the parents' peripheral blood for exome sequencing analysis. Pathogenic variants identified would then be validated in family members. Based on the definitive genetic diagnosis and comprehensive counseling, the couple opted for termination of the pregnancy. The confirmation of the fetus as male was critical to this decision, as *L1CAM*‐associated hydrocephalus is an X‐linked recessive condition, and a female fetus would be an unaffected carrier. This finding provided a clear etiology for the recurrent condition, enabling the family to pursue Preimplantation Genetic Testing (PGT) for future pregnancies. To contribute this clinically significant information to the public domain, the variant has been submitted to the ClinVar database (Accession: VCV003573920.2).

### 2.3 Exome Sequencing

Genomic DNA was extracted from the amniotic fluid and whole blood of both parents. Maternal cell contamination in the amniotic fluid sample was ruled out by short tandem repeat (STR) analysis. Exome capture was carried out with Illumina's Nextera Rapid Capture Exome Kit. More than 10G raw data were obtained, resulting in a coverage of at least 100×.

### 2.4 Data Analysis

Sequence reads were mapped to the human genome (hg19) using standard ES analysis methods. The *L1CAM* reference sequence used was NM_000425.5 from GenBank. A CNV detection pipeline was performed with ExomeDepth software, focusing on virtual gene panels associated with fetal lateral ventricle dilation, unclear septum pellucidum, hydrocephalus, and agenesis of the corpus callosum.

### 2.5 Variant Classification and Pathogenicity Prediction

The pathogenicity of the rare variants identified by ES was classified by the guidelines of ACMG (Richards et al. 2015). SIFT, PolyPhen2, REVEL and other prediction tools were included (Ioannidis et al. 2016; Schwarz et al. 2014; Shihab et al. 2013). For those missense variants classified as VUS according to ACMG guidelines, we additionally employed AlphaMissense (Cheng et al. 2023; Tordai et al. 2024) for pathogenicity prediction. AlphaMissense generates a score from 0 to 1, where a higher score indicates a higher probability of pathogenicity. Following the tool's original recommendation, variants with a score > 0.564 were considered ‘likely pathogenic’.

### 2.6 Validation With Sanger Sequencing

Sanger sequencing was used to confirm the rare variants identified by trio ES. Primers for the target region of the variants were designed using Primer3 online software (http://primer3.ut.ee/).

### 2.7 Protein Structure Modeling With AlphaFold

To predict the structural impact of the p.His410Asp variant, 3D models for both the wild‐type and mutant *L1CAM* protein sequences were generated. Both sequences were submitted to the Google DeepMind AlphaFold Server, which utilizes the AlphaFold 3 model. For each sequence, the top‐ranked model based on pLDDT scores was selected for subsequent structural analysis (https://alphafoldserver.com/) (Abramson et al. 2024).

## 3 Results

### 3.1 Clinical Evaluation Results

The MRI results of the two maternal uncles showed agenesis of the corpus callosum, with the body of the lateral ventricles parallel and separated, narrow frontal horns, noticeably enlarged atrial and occipital horns, an enlarged and upward displaced third ventricle, and the corpus callosum not visualized (see Figure 1e–j).

### 3.2 ES Results

The ES results showed that no pathogenic CNVs were found, nor were there any variants classified as ‘pathogenic/likely pathogenic.’ However, several VUS were identified, as shown in Table S1. A total of four variants were classified as VUS. After further evaluation using AlphaMissense, only one variant, *L1CAM*, was predicted to be likely pathogenic (LP), with a score of 0.94, while the others were assessed as benign.

### 3.3 Sanger Sequencing Verification Results

**FIGURE 2:**
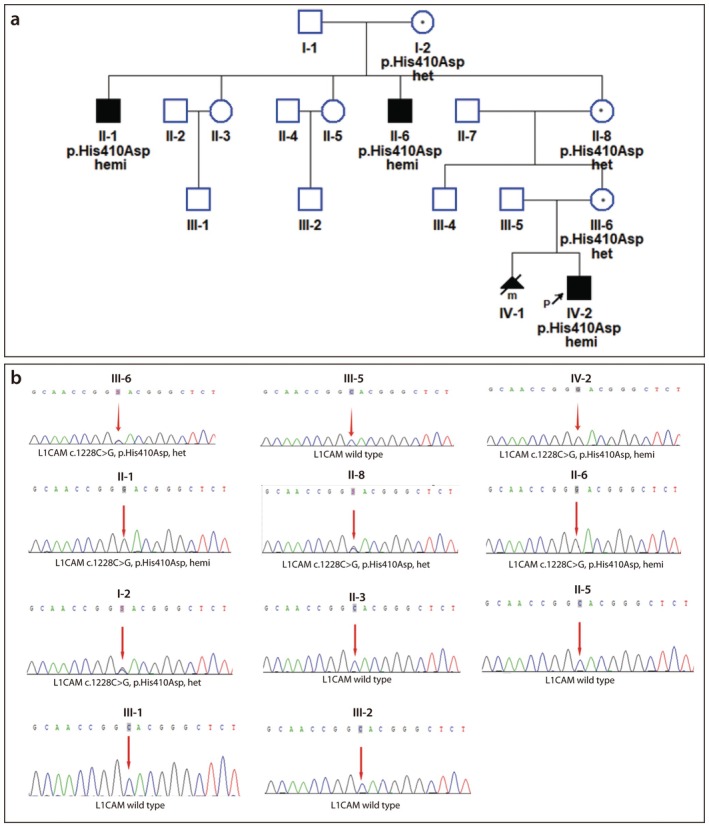
Co‐segregation analysis of the *L1CAM* p.His410Asp variant. (a) Family pedigree showing X‐linked inheritance. The p.His410Asp variant co‐segregates with the disease phenotype. Standard symbols are used; the arrow indicates the proband. (b) Representative Sanger sequencing chromatograms showing the c.1228C>G variant in an affected hemizygous male, a heterozygous female carrier, and a wild‐type relative.

The p.His410Asp variant was validated through Sanger sequencing in all maternal relatives. The results showed that the mother and grandmother of the pregnant woman were both heterozygous, while both uncles were hemizygous. Two aunts and the sons of the aunts were found to be wild type (see Figure 2b).

We have redrawn the pedigree chart (see Figure 2a), which demonstrates that the p.His410Asp variant co‐segregates with the disease in the family. According to the ACMG guidelines, the co‐segregation evidence (PP1) was upgraded to PP_Strong, and the p.His410Asp variant has been upgraded from VUS to Likely pathogenic.

### 3.4 Protein Structure Modeling

**FIGURE 3:**
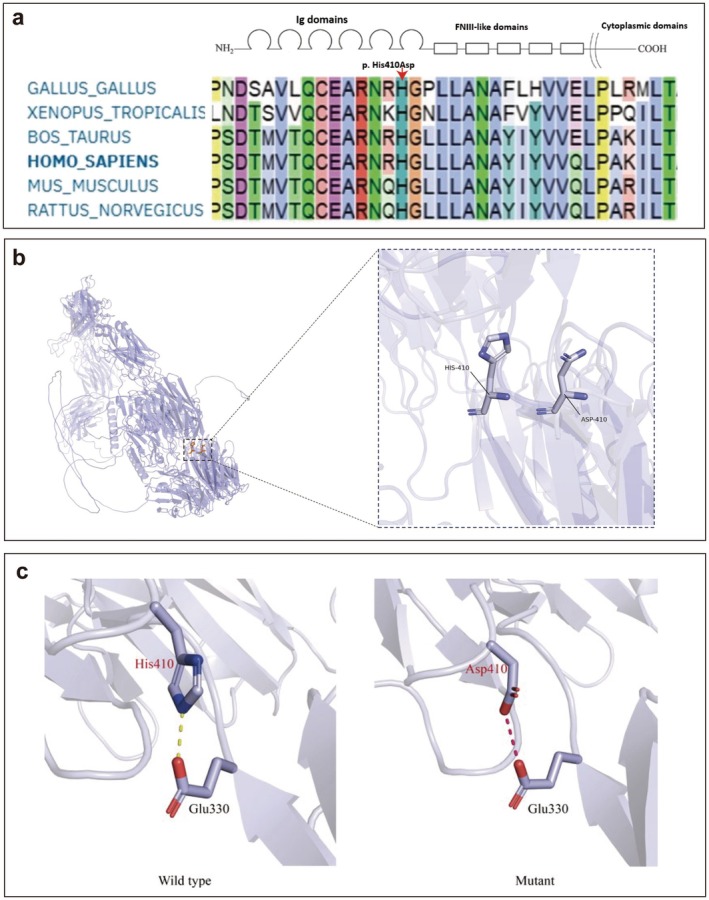
Structural impact of the p.His410Asp variant. (a) The His410 residue (red box) is highly conserved across multiple vertebrate species. (b) Structural models of the wild‐type (His410) and mutant (Asp410) protein regions, generated by the AlphaFold 3 server. (c) The p.His410Asp mutation is predicted to disrupt a stabilizing salt bridge (dashed line) that exists between His410 and Glu330 in the wild‐type structure, likely decreasing protein stability.

Multispecies alignment of the *L1CAM* sequence and protein domain analysis revealed that the p.His410 residue is highly conserved through evolution (Figure 3a). Figure 3b illustrates the structural changes resulting from the His410Asp variant in the *L1CAM* protein, as predicted by AlphaFold. In the wild‐type protein, the histidine residue at position 410 (His‐410) has a specific orientation and forms hydrogen bonds via its imidazole ring. Following the mutation to aspartic acid (Asp‐410), notable alterations emerge. The carboxyl side chain of aspartic acid introduces new polar interactions, potentially altering the local electrostatic environment of the protein. Figure 3c shows the interaction between His410 and Glu330; in the wild‐type protein, a salt bridge forms between these residues, stabilizing local interactions. However, the mutation to Asp410 disrupts this salt bridge, resulting in electrostatic repulsion. It is hypothesized that this variant may decrease protein stability.

These changes may have downstream effects, including alterations in folding stability, protein dynamics, and interaction capabilities with other biomolecules. This comparison highlights a shift in side‐chain characteristics and potentially in overall protein conformation, which could affect the functional properties of *L1CAM*.

## 4 Discussion

Identifying pathogenic variants is crucial for individuals with a family history of genetic disorders. Traditionally, identifying pathogenic variants involves Trio ES of the proband, followed by Sanger sequencing to confirm variants in other family members. However, this approach often faces challenges, particularly when the proband does not show easily identifiable variants, such as frame‐shift, splice‐site, or nonsense variants (He et al. 2023; Kanemura et al. 2005; Rehnberg et al. 2011; Silan et al. 2005; Zhou et al. 2022). Many novel missense variants lack prior documentation, leading to their classification as VUS under ACMG guidelines. When numerous VUS are present, it becomes increasingly difficult to determine whether a true pathogenic variant exists among them.

Our rationale for using AlphaMissense in this study was primarily strategic, designed to overcome the common challenge of interpreting multiple VUSs from exome sequencing. In a clinical context, especially in large families, validating every VUS with Sanger sequencing is impractical due to time and cost. Therefore, we employed AlphaMissense not as a direct line of evidence for pathogenicity, but as a highly effective prioritization tool. Its demonstrated high accuracy, which integrates structural and evolutionary data, allowed us to reliably filter the VUS list and identify the single most promising candidate for follow‐up. This approach enabled us to focus our resources efficiently. The definitive reclassification of the variant from VUS to Likely Pathogenic was achieved exclusively through the subsequent Sanger sequencing, which confirmed its co‐segregation with the disease in the family, thereby satisfying a key ACMG criterion. In large families, validating all VUS through Sanger sequencing can be time consuming, and performing ES for all members can be prohibitively expensive. Our study highlights the usefulness of AlphaMissense as a preliminary filtering tool that retains only those variants predicted to be likely pathogenic for further Sanger sequencing validation. This strategy improves the efficiency of the validation process, saving both time and costs. AlphaMissense serves as a valuable initial screening tool in clinical settings.

Additionally, using AlphaFold to predict protein structures provides further evidence supporting the pathogenicity of VUSs. By employing these advanced computational tools, we can better understand the functional implications of genetic variants, enhancing our ability to provide accurate diagnoses and effective counseling.

Overall, integrating rapidly evolving artificial intelligence technologies into clinical genetic testing marks a significant advancement in medical genetics. By leveraging tools like AlphaMissense and AlphaFold, we can improve the accuracy and efficiency of variant assessment, ultimately leading to better patient outcomes and advancing genetic diagnostics.

## 5 Conclusion

In conclusion, this study underscores the critical role of advanced computational tools, such as AlphaMissense and AlphaFold, in the identification and validation of pathogenic variants, particularly in families with a history of hydrocephalus linked to the *L1CAM* gene. By efficiently narrowing down variants of uncertain significance and confirming likely pathogenic mutations, we enhanced the diagnostic process and provided clearer insights into genetic risks. The integration of artificial intelligence into clinical genetics not only streamlines variant assessment but also paves the way for improved prenatal diagnostics and better management strategies for at‐risk families.

## Author Contributions

Zhihui Wang was responsible for writing the article. Xuna Shen served as the attending physician for the patients in this study. Chendi Teng handled fetal ultrasound imaging and MRI. Chenyang Xu conducted genetic testing and bioinformatics analysis. Rongyue Wang oversaw the family recall and clinical examination. Yanbin He and Weiyan Wu oversaw the construction of the AlphaFold modeling and protein structure analysis. Xutao Hong contributed ideas, managed the manuscript submission, and was responsible for the overall study. All authors contributed to the study design, data collection, analysis, and manuscript preparation, and no outside funding was received for this research. All authors read and approved the final manuscript.

## Funding

The study is supported by a grant from the Basic Scientific Research Projects in Wenzhou City (Y2023010).

## Ethics Statement

The studies involving human participants were reviewed and approved by the Ethics Committee of Wenzhou Central Hospital (Approval Number: 2023‐022). This study has been conducted in accordance with recognized ethical standards, including the Declaration of Helsinki, which outlines principles for human research, as well as relevant guidelines for good clinical practice. Written informed consent to participate in this study was obtained from the legal guardians or next of kin of all participants prior to their inclusion. Additionally, written informed consent for the publication of any potentially identifiable images or data included in this article was secured from the legal guardians or next of kin of any individuals and minors involved. The study ensures that the rights and welfare of all participants were prioritized throughout the research process.

## Consent

Written informed consent was obtained from all participants prior to their inclusion in the study.

## Conflicts of Interest

The authors declare no conflicts of interest.

## Supporting information


**Table S1:** List of VUS variants.

## Data Availability

The data presented in the study are deposited in the ClinVar repository, Accession: SCV005627753. The data that support the findings of this study are available from the corresponding author upon reasonable request.
