# Supplementary material for: Accelerated Identification and Preliminary Validation of a Pathogenic Missense Variant in the L1CAM Gene in a Pregnant Woman With Sonographic Anomalies Using AlphaMissense
Source: Mol Genet Genomic Med. 2025 Dec 21;13(12):e70169. doi: 10.1002/mgg3.70169 (PMC12719231; doi:10.1002/mgg3.70169)
Supplement: Supplementary file 1 — Table S1: List of VUS variants. [file MGG3-13-e70169-s001.docx]

**Supplemental Table** 1. List of VUS variants

| gene | position | OMIM | RefSeq transcript | Variant | Het/Hom/Hem |  | ACMG criteria | AlphaMissense pathogenicity score | AlphaMissense pathogenicity class |
| --- | --- | --- | --- | --- | --- | --- | --- | --- | --- |
| QARS1 | 3:49136944 | 603727 | NM_005051.3 | c.1525C>T, p.Arg509Trp | het | paternal | VUS:PM2+PP3 | 0.1682 | Likely benign |
| DAG1 | 3:49569899 | 128239 | NM_004393.6 | c.1955G>A, p.Arg652Gln | het | paternal | VUS:PM2+PP3 | 0.0837 | Likely benign |
| L1CAM | X:153135014 | 308840 | NM_001278116.2 | c.1228C>G, p.His410Asp | hemi | maternal | VUS:PM2+PP3+PP4 | 0.93 | Likely pathogenic |
| ASPM | 1:197115498 | 605481 | NM_018136.5 | c.70C>G, p.Leu24Val | het | maternal | VUS:PM2+PP3 | 0.081 | Likely benign |
| SKI | 1:2235497 | 164780 | NM_003036.4 | c.1430A>G, p.Asp477Gly | het | De novo | VUS:PM2+PM6+PP3 | 0.086 | Likely benign |

Detected gene variants associated with "agenesis of the corpus callosum and hydrocephalus," classified as VUS by the ACMG. Predictions made using AlphaMissense indicate that only one variant in *L1CAM* is classified as "likely pathogenic". PP3 evidence means that the PolyPhen-2 and SIFT prediction tools indicate a "D" (damaging) classification.
